# Comparing Genomic Profiles of *ALK* Fusion-Positive and *ALK* Fusion-Negative Nonsmall Cell Lung Cancer Patients

**DOI:** 10.1055/s-0044-1787301

**Published:** 2024-06-13

**Authors:** Wenchao Xia, Jing Yang, Hongbin Li, Ling Li, Jinfeng Liu

**Affiliations:** 1 Department of Thoracic Surgery, Tianjin Chest Hospital, Tianjin, People's Republic of China; 2 Department of Pathogenic Biology, Logistics University of Chinese People's Armed Police Force, Tianjin, People's Republic of China; 3 Department of Oncology, Rongcheng County People's Hospital, Baoding, People's Republic of China; 4 Department of Medicine, Yinfeng Gene Technology Co., Ltd., Jinan, People's Republic of China; 5 Department of Thoracic Surgery, The First Hospital of Hebei Medical University, Shijiazhuang, People's Republic of China

**Keywords:** NSCLC, *ALK* fusion, next-generation sequencing, genomic landscape

## Abstract

**Background** Anaplastic lymphoma kinase ( *ALK* ) fusion events account for 3 to 7% of genetic alterations in patients with nonsmall cell lung cancer (NSCLC). This study aimed to explore the landscape of *ALK* fusion-positive and *ALK* fusion-negative in a large cohort of NSCLC patients.

**Methods** The formalin-fixed paraffin-embedded specimens of NSCLC patients who underwent next-generation sequencing from 2020 to 2023 in Yinfeng Gene Technology Co., Ltd. Clinical laboratory were included in this study.

**Results** In the current study, a total of 180 (3.20%) patients tested positive for *ALK* fusions in 5,622 NSCLC samples. Within the *ALK* -positive cohort, a total of 228 *ALK* fusions were identified. Furthermore, five novel *ALK* fusion partners, including *DAB1-ALK* , *KCMF1-ALK* , *KIF13A-ALK* , *LOC643770-ALK* , and *XDH-ALK* were identified. In cases with *ALK* fusion-positive, *TP53* alterations were the most prevalent (26.3%), followed by *CDKN2A* (8.4%), epidermal growth factor receptor ( *EGFR* , 5.6%), and *ALK* (5.6%). By contrast, *EGFR* alterations were most prevalent (51%) in patients with *ALK* fusion-negative NSCLC, followed by *TP53* (42.7%), *KRAS* (11.6%), and *CDKN2A* (11.3%). A total of 10 cases where *ALK* fusion co-occurred with *EGFR* mutations were also identified. Notably, the *ALK* fusion positivity rate was higher in younger patients ( *p* < 0.0001) and in female patients ( *p* = 0.0429). Additionally, positive *ALK* test results were more prevalent in patients with high programmed death-ligand 1 expression, especially when applying a 50% cutoff.

**Conclusions** Collectively, these findings offer valuable genomic insights that could inform the personalized clinical care of patients with NSCLC harboring *ALK* fusions within the context of precision medicine.

## Introduction

Lung cancer is the main contributor to cancer-related mortality across the globe, with nonsmall cell lung cancer (NSCLC) encompassing >80% of all diagnosed cases. 1 2 Within the population of patients with NSCLC, an estimated 2 to 7% exhibit anaplastic lymphoma kinase ( *ALK* ) gene rearrangements, resulting in the abnormal expression and oncogenic activation of *ALK* . 3 4 The most prevalent and canonical *ALK* gene arrangement in NSCLC is the echinoderm microtubule-associated protein-like 4 ( *EML4* )- *ALK* fusion, wherein various *EML4* breakpoints fuse in-frame with the kinase domain of *ALK* . 5 Notably, >15 distinct *EML4-ALK* fusion variants have been identified in NSCLC, with v1, v2, and v3a/b being the most frequently encountered variants. 6 In addition to these, certain *ALK* fusions, although less prevalent in NSCLC, have been reported, classified as noncanonical *ALK* fusions. These include kinesin family member 5B ( *KIF5B* )- *ALK* , *TRK* -fused gene ( *TFG* )- *ALK* , kinesin light chain 1 ( *KLC1* )- *ALK* , striatin ( *STRN* )- *ALK* , and *TNFAIP3* interacting protein 2 ( *TNIP2* )- *ALK* . 7 8 9 10 It is noteworthy that some *ALK* fusions are predominantly found in other types of cancers.

In recent years, numerous clinical trials have been conducted to explore treatments targeting specific molecular mechanisms, such as *ALK* fusion. Small molecule inhibitors designed for *ALK* fusion, including crizotinib, alectinib, brigatinib, and lorlatinib, have been approved by the U.S. Food and Drug Administration for various cancer types. 11 12 13 Despite these advancements, it is noteworthy that a subset of patients with *ALK* -positive NSCLC (10–40%) fail to respond to ALK tyrosine kinase inhibitors (TKIs). This emphasizes the clinical importance of further stratifying patients with *ALK* -positive NSCLC based on their response to TKIs. While there is potential benefit in identifying *ALK* fusions, it remains uncertain whether tumors with *ALK* fusions constitute a distinct, albeit rare, subtype that should be detected early for targeted therapy. With the continued development of next-generation sequencing (NGS) technologies, obtaining the genomic landscape of patients with cancer has become more affordable and accessible. In the current study, the aim was to unveil the genomic landscape of *ALK* fusion-positive tumors in 180 NSCLC patients who underwent sequencing. The aim was to elucidate their genomic mutation patterns and characteristics, which could notably contribute to the development of more precise and effective treatment strategies.

## Materials and Methods

### Clinical Specimens

The formalin-fixed paraffin-embedded (FFPE) specimens of NSCLC patients who underwent NGS from 2020 to 2023 in Yinfeng Gene Technology Co., Ltd. were included. The diagnosis of the specimens was confirmed by hematoxylin and eosin staining by an independent pathologist. The specimens were required to have a percentage of tumor cells over 20% and a size ≥1 mm for further analysis.

### DNA Extraction and Next-Generation Sequencing

The DNA extraction process involved a microdissection technique for precise dissection of tissue blocks. Genomic DNA was then extracted from FFPE samples using the QIAamp DNA FFPE Tissue Kits (Qiagen GmbH, 56404). The quality of the isolated genomic DNA was assessed through the measurement of DNA concentration, using Qubit DNA Assay Kits and a Qubit 2.0 Fluorometer (Thermo Fisher Scientific, Inc.), as well as through 1% agarose gel electrophoresis to evaluate DNA degradation. To create DNA fragments in the range of 180 to 280 bp hydrodynamic shearing was executed on 0.6 g of genomic DNA using the M220 Focused-ultrasonicator (Covaris, LLC). Subsequently, sequencing libraries were prepared according to the manufacturer's instructions, employing the Agilent SureSelect Human All Exon V6 kit (Agilent Technologies, Inc. 5190-8863). For the purpose of target enrichment, the constructed libraries were hybridized with custom-designed biotinylated oligonucleotide probes (Roche Diagnostics). Following this, the index-coded library samples were clustered using the Illumina cBot Cluster Generation System (Illumina, Inc.) and the DNA libraries were sequenced with the use of an Illumina HiSeq 2000 system (Illumina, Inc.).

### Statistical Analysis

In the current study, the association of *ALK* fusions with age and sex was examined through the Fisher's exact test and the Mann–Whitney U test. Furthermore, the relationship between genomic characteristics and the proportion of programmed death-ligand 1 (PD-L1) expression was assessed using the Fisher's exact test with the cutoff values set at 1 and 50%. It is noteworthy that all statistical tests were conducted as two-sided tests. *p* < 0.05 was considered to indicate a statistically significant difference.

## Results

### Characteristics of Patients with *ALK* Fusions

A total of 5,622 patients diagnosed with NSCLC who had undergone tissue-based NGS with 500 cancer gene panel were included in the current analysis. Among them, 180 (3.2%) patients were identified as having *ALK* fusions. The median age of the patients was 58 years, with an age range 26 to 82; 48% of the patients were men. The majority of patients had lung adenocarcinoma (177 cases; 98.3%), whereas a small number had lung squamous cell carcinoma (two cases; 1.1%) and lung adenosquamous carcinoma (one case; 0.6%). Among the 180 patients with *ALK* fusions, 65 (36.1%) patients had evaluable microsatellite instability (MSI) status, and no patients with high MSI were detected. Furthermore, 54 (30%) patients had evaluable tumor mutational burden (TMB) status, with only two (1.1%) patients presenting with high TMB ( Table 1 ). The cutoff for MSI was 29 (13.5%) based on the evaluation of 55 microsatellite markers, and the cutoff for TMB was nine mutations per megabase for the panels. Notably, NGS was conducted on 180 pairs of tumor and white blood cell samples, and all samples that passed the histology quality control yielded sufficient DNA quantities for NGS analysis.

**Table 1:** Clinicopathological information of the *ALK* fusion nonsmall cell lung cancer patients

Characteristics	All patients ( *N* = 180)
Sex, *n* (%)	
Male	87 (48.3%)
Female	93 (51.7%)
Age, median (range)	58 (26–82)
Clinical stages	
I/II	42 (23.3%)
III	29 (16.1%)
IV	109 (60.6%)
MSI status, *n* (%)	
MSI-H	0 (0%)
MSS	65 (36.1%)
N/A	115 (63.9%)
TMB status, *n* (%)	
TMB-H	2 (1.1%)
TMB-L	52 (28.9%)
N/A	126 (70%)
Cancer type, *n* (%)	
Lung adenocarcinoma	177 (98.3%)
Lung squamous cell carcinoma	2 (1.1%)
Lung adenosquamous carcinoma	1 (0.6%)

Abbreviations: H, high; L, low; MSI, microsatellite instability; MSS, microsatellite stable; N/A, not applicable; TMB, tumor mutational burden.

### Molecular Features of *ALK* Fusion-Positive Patients

**Fig. 1:**
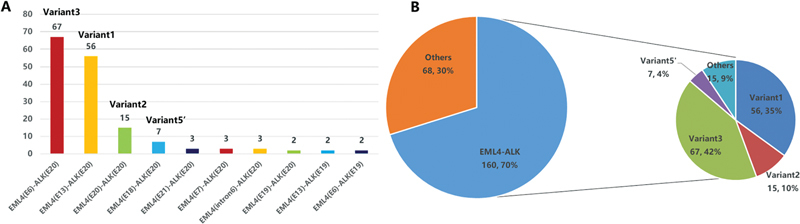
Mutational proﬁles and partners of *ALK* fusion-positive patients. ( **A** ) The statistics of different *EML4-ALK* rearrangement forms. ( **B** ) Distribution of *ALK* fusion partners and *EML4-ALK* variants.

Using NGS, a total of 180 patients who harbored *ALK* fusions were identified and categorized into the *ALK* fusion-positive group, whereas those without *ALK* fusions constituted the *ALK* fusion-negative group. Among the ALK cohort, although a total of 228 ALK fusions were identified, 43 (25%) patients exhibited simultaneous occurrences of ≥2 distinct ALK fusions ( Table 2 ). The predominant fusion partner within the present cohort was *EML4* , accounting for 70% (160/228) of cases followed by *KLC1* (2.2%; 5/228), *KIF5B* (2.2%; 5/228), and *HIP1* (0.9%; 2/228). In the *ALK* fusion-positive patients, various *EML4-ALK* variants were identified, including variant 1 (E13; A20; 35.0%; 56/160), variant 2 (E20; A20; 9.4%; 15/160), variant 3 (E6; A20; 41.8%; 67/160), variant 5 (E2; A20; 4.4%; 7/160), and other variants (9.4%; 15/160; Fig. 1A, B ).

**Table 2:** The co-occurrence of *ALK* fusions observed in 43 patients

Patient (43)	Fusion site 1	Fusion site 2	Fusion site 3	Fusion site 4	Number
Pt1	*EML4-ALK* (exon13: exon20)	*ALK-FAM179A* (intergenic) (exon19: intergenic)	–	–	2
Pt2	*HIP1-ALK* (exon30: exon19)	*ALK-CUX1* (exon19: exon3)	–	–	2
Pt3	*ALK* -LOC100996478 (exon19: exon1)	*KCMF1-ALK* (exon2: exon20)	–	–	2
Pt4	*EML4-ALK* (exon6: exon20)	*ALK-LCLAT1* (exon19: promoter)	–	–	2
Pt5	*EML4-ALK* (exon13: exon20)	*ALK-TANK* (exon19: exon3)	–	–	2
Pt6	*EML4-ALK* (exon13: exon20)	*ALK-PDE5A* (exon18: exon21)	–	–	2
Pt7	*ALK-KLHL1* (intergenic) (exon19: intergenic)	*EML4-ALK* (exon6: exon20)	–	–	2
Pt8	*ALK-CTNNA2* (intergenic) (exon7: intergenic)	*EML4-ALK* (exon6: exon20)	–	–	2
Pt9	*EML4-ALK* (exon13: exon20)	*ALK-CDC42EP3* (exon19: promoter)	–	–	2
Pt10	*ALK-HS1BP3* (exon19: promoter)	*EML4-ALK* (exon20: exon20)	–	–	2
Pt11	*EML4-ALK* (exon13: exon20)	*ALK-SLC8A1* (intergenic) (exon19: intergenic)	–	–	2
Pt12	*EML4-ALK* (exon13: exon20)	*ALK-ITSN2* (exon19: exon2)	–	–	2
Pt13	*EML4-ALK* (exon13: exon20)	*ALK-CREB1* (exon19: exon1)	–	–	2
Pt14	*ALK-TRMT61B* (exon19: exon4)	*EML4-ALK* (exon20: exon20)	*ALK-IGK* (exon19: exon0)	–	3
Pt15	*EML4-ALK* (exon13: exon20)	*ALK-RTKN* (exon19: exon2)	–	–	2
Pt16	*EML4-ALK* (exon13: exon20)	*XDH-ALK* (exon23: exon19)	*ALK-SPAG16* (exon19: exon10)	*ALK-PRKD3* (exon18: exon11)	4
Pt17	*ALK* -LOC100996478 (exon19: promoter)	*EML4-ALK* (exon6: exon20)	–	–	2
Pt18	*EML4-ALK* (exon13: exon20)	*ALK-SUPT7L* (exon19: exon6)	–	–	2
Pt19	*EML4-ALK* (exon20 exon20)	*ALK-SLIT2* (intergenic) (exon13: intergenic)	–	–	2
Pt20	*ALK-NTRK2* (intergenic) (exon19: intergenic)	*HIP1-ALK* (exon28: exon20)	–	–	2
Pt21	*ALK-COX7A2* L (intergenic) (exon19: intergenic)	*EML4-ALK* (exon6: exon20)	–	–	2
Pt22	*KLC1-ALK* (exon9: exon20)	*ALK-XRCC3* (exon19: exon6)	–	–	2
Pt23	*ALK-PRKCE* (exon19: exon9)	*ALK-GALNT14* (exon19: exon10)	*EML4-ALK* (exon20: exon20)	–	3
Pt24	*EML4-ALK* (exon6: exon20)	*ALK-MAP4K3* (exon19: exon3)	–	–	2
Pt25	*EML4-ALK* (exon13: exon20)	*ALK-PRKCE* (exon19: exon10)	–	–	2
Pt26	*KIF13A-ALK* (exon19: exon20)	*ALK-MAN1A1* (intergenic) (exon19: intergenic)	–	–	2
Pt27	*EML4-ALK* (exon6: exon20)	*ALK-LINC00301* (exon20: exon6)	–	–	2
Pt28	*EML4-ALK* (exon13: exon20)	*ALK-TACR3* (intergenic) (exon19: intergenic)	–	–	2
Pt29	*ALK-VRK2* (intergenic) (exon19: intergenic)	*EML4-ALK* (exon6: exon20)	–	–	2
Pt30	*ALK-SYNE2* (exon19: exon24)	*EML4-ALK* (exon6: exon20)	–	–	2
Pt31	*KLC1-ALK* (exon9: exon20)	*ALK-DPP10* (exon19: exon2)	–	–	2
Pt32	*EML4-ALK* (exon6: exon20)	*ALK-MAP4K3* (exon19: exon14)	–	–	2
Pt33	*EML4-ALK* (exon21: exon20)	*ALK-TBC1D8B* (exon19: exon19)	–	–	2
Pt34	*ALK-GCFC2* (exon19: promoter)	*EML4-ALK* (exon6: exon20)	–	–	2
Pt35	*EML4-ALK* (exon18: exon20)	*ALK-CRIM1* (intergenic) (exon19: intergenic)	–	–	2
Pt36	*EML4-ALK* (exon13: exon20)	*ALK-COX7A2L* (exon19: exon2)	–	–	2
Pt37	*ALK-GTDC1* (exon19: exon4)	*EML4-ALK* (exon13: exon20)	–	–	2
Pt38	*ALK-BCL11A* (exon19: exon3)	*EML4-ALK* (exon13: exon20)	–	–	2
Pt39	*ALK-PRKCE* (exon19: exon11)	*EML4-ALK* (exon6: exon20)	–	–	2
Pt40	*ALK-BRE* (exon19: exon4)	*EML4-ALK* (exon6: exon20)	–	–	2
Pt41	*CLIP4* (intergenic) *-ALK* (intergenic: exon14)	*ALK-* C2orf91 (intergenic) (exon19: intergenic)	*EML4-ALK* (exon6: exon20)	–	3
Pt42	ALK-AK4 (intergenic) (exon19: intergenic)	*EML4-ALK* (exon6: exon20)	–	–	2
Pt43	*MTA3* (intergenic) *-ALK* (intergenic: exon20)	*EML4-ALK* (exon6: exon20)	–	–	2

In addition to *EML4-ALK* fusions, the cohort of the current study also revealed the presence of other *ALK* fusion partners. It is noteworthy that novel variants, including intergenic fusions, have been extensively analyzed due to their potential marked clinical implications for fusion carriers. 14 Consequently, these novel variants, particularly intergenic fusions, have garnered considerable attention as they may represent potential targetable variants. In the present study, a total of five *ALK* intergenic fusions were identified ( Table 3 ). Typically, fusions involving intergenic regions were considered unlikely to produce functional fusion transcripts. However, emerging evidence suggests that intergenic fusions may also lead to the generation of functional fusion proteins after transcription, potentially involving mechanisms such as chromothripsis and alternative splicing. 14 For five cases where RNA-based NGS assays were unsuccessful due to limited materials, further investigation is warranted. Furthermore, among these cases, it is noteworthy that two patients, *CLIP4* (intergenic)- *ALK* (intergenic, exon14) and *MTA3* (intergenic)- *ALK* (intergenic, exon20), also carried the canonical *EML4-ALK* fusion (exon6; exon20; Table 3 ).

**Table 3:** The other *ALK* fusion partners observed in our cohort except for *EML4-ALK*

	*ALK* fusion partner	Fusion site 1	Number	Fusion site 2
*ALK* intergenic fusion	*PCDH9* (intergenic)	*PCDH9* (intergenic)- *ALK* (intergenic: exon20)	1	–
	*CLIP4* (intergenic)	*CLIP4* (intergenic)- *ALK* (intergenic: exon14)	1	*EML4-ALK* (exon6: exon20)
	*MIR1973* (intergenic)	*MIR1973* (intergenic)- *ALK* (intergenic: exon19)	1	–
	*MTA3* (intergenic)	*MTA3* (intergenic)- *ALK* (intergenic: exon20)	1	*EML4-ALK* (exon6: exon20)
	*YPEL5* (intergenic)	*YPEL5* (intergenic)- *ALK* (intergenic: exon1)	1	–
Known *ALK* fusion	*KLC1*	*KLC1-ALK* (exon9: exon20)	5	–
	*HIP1*	*HIP1-ALK* (exon30: exon19)	1	–
	*HIP1*	*HIP1-ALK* (exon28: exon20)	1	–
	*KIF5B*	*KIF5B-ALK* (exon24: exon20)	3	–
	*KIF5B*	*KIF5B-ALK* (exon17: exon20)	1	–
	*KIF5B*	*KIF5B-ALK* (exon18: exon20)	1	–
Novel *ALK* fusion	*KCMF1*	*KCMF1-ALK* (exon2: exon20)	1	–
	*XDH*	*XDH-ALK* (exon23: exon19)	1	*EML4-ALK* (exon13: exon20)
	*KIF13A*	*KIF13A-ALK* (exon19: exon20)	1	–
	*LOC643770*	*LOC643770-ALK* (exon1: exon20)	1	–
	*DAB1*	*DAB1-ALK* (exon3: exon20)	1	–

**Fig. 2:**
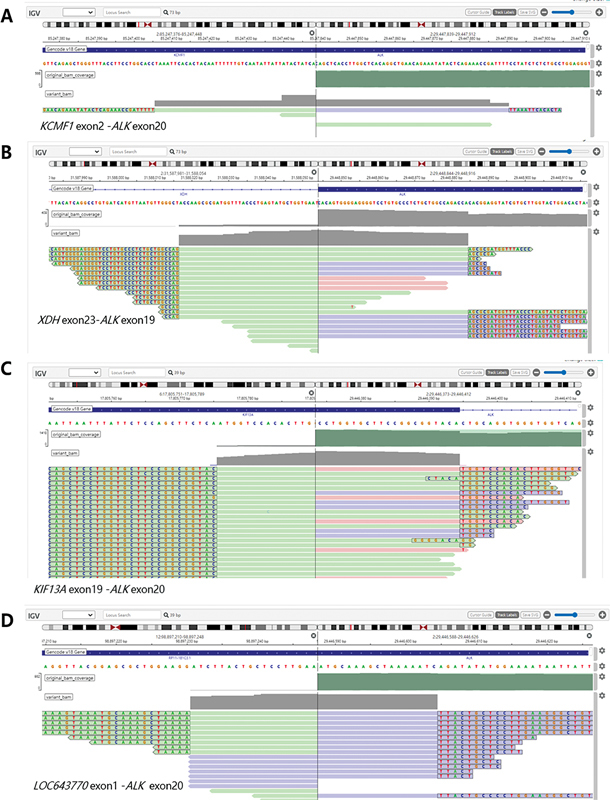
The novel *ALK* fusions were examined on Integrative Genomic Viewer (IGV) software. ( **A** ) *KCMF1* -ALK (exon2: exon20). ( **B** ) *XDH-ALK* (exon23: exon19). ( **C** ) *KIF13A-ALK* (exon19: exon20). ( **D** ) *LOC643770-ALK* (exon1: exon20).

**Fig. 3:**
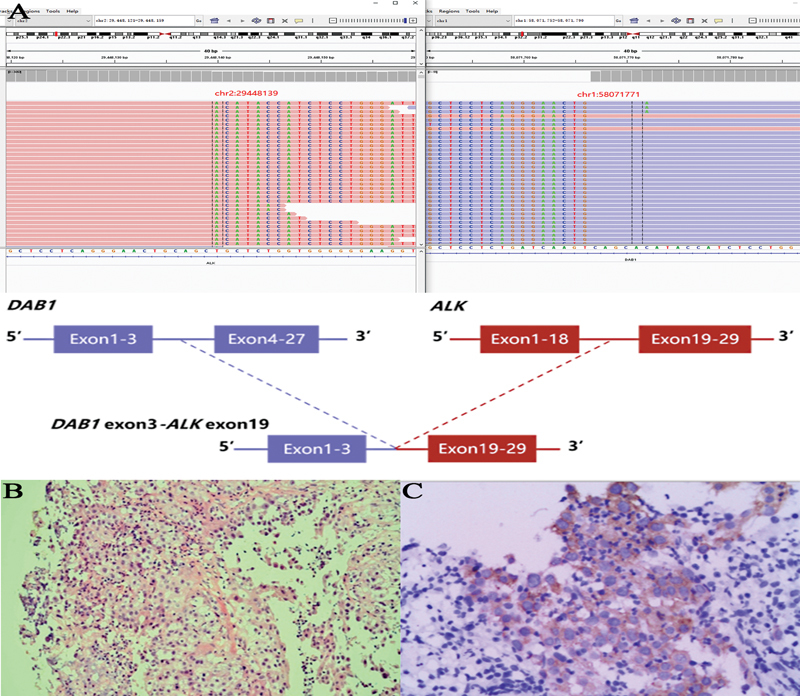
Diagrammatic sketch of the *ALK* fusion of the patient. ( **A** ) *ALK* fusion. ( **B** ) Pathology. ( **C** ) Representative images of immunohistochemical (IHC) staining for *ALK* of the patient.

Additionally, five novel *ALK* fusion partners were identified ( Table 3 ). These novel fusions included *KCMF1-ALK* ( Fig. 2A ), *XDH-ALK* ( Fig. 2B ), *KIF13A-ALK* ( Fig. 2C ), *LOC643770-ALK* ( Fig. 2D ), and *DAB1-ALK* ( Fig. 3A ). Notably, one patient with an *XDH-ALK* fusion (exon23; exon19) was found to concurrently possess the canonical *EML4-ALK* fusion (exon13; exon20; Table 3 ). However, a previous study 15 reported that patients with NSCLC and complex *ALK* fusions could potentially have better treatment outcomes to *ALK* TKI therapy. Furthermore, a novel *DAB1-ALK* fusion variant was identified in a patient with pulmonary adenosquamous carcinoma ( Fig. 3A ). To the best of our knowledge, the present study is the first to report a *DAB1-ALK* fusion in patients with NSCLC. To confirm the presence of the *ALK* fusion, pathology and immunohistochemistry (IHC) were performed on puncture tissue samples. The results validated the existence of the *ALK* fusion in adenosquamous carcinoma samples ( Fig. 3B, C ).

### Differences of Mutant Genes between Patients with *ALK* Fusion-Positive and Patients with *ALK* Fusion-Negative Nonsmall Cell Lung Cancer

**Fig. 4:**
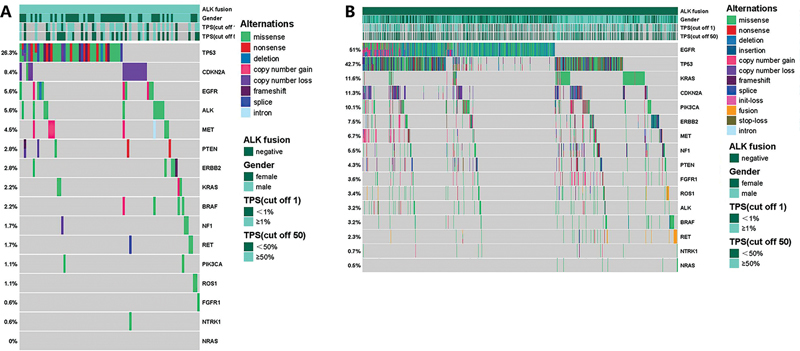
Differences of mutant genes between *ALK* fusion- positive group and *ALK* fusion-negative group in nonsmall cell lung cancer (NSCLC). ( **A** ) Mutational proﬁles of *ALK* fusion- positive NSCLC patients in our study. ( **B** ) Mutational proﬁles of *ALK* fusion-negative NSCLC patients in our study. The genes are ranked by the frequency of the mutations across all samples.

In the present analysis of 180 *ALK* fusion-positive cases, *TP53* alterations were the most prevalent (26.3%), followed by *CDKN2A* (8.4%), epidermal growth factor receptor ( *EGFR* , 5.6%), and *ALK* (5.6%). Other noteworthy genomic alterations include *MET* (4.5%), *PTEN* (2.8%), *ERBB2* (2.8%), *KRAS* (2.2%), and *BRAF* (2.2%). Among the ALK fusion-positive cases, a total of 55 variations were identified in *TP53* , encompassing 30 missense mutations, 10 nonsense mutations, five splice mutations, five frameshift mutations, four copy number loss mutations, and one deletion mutation. Additionally, patients with ALK fusion-positive NSCLC exhibited 15 variations in *CDKN2A* , including 14 copy number loss mutations and one missense mutation. Moreover, *MET* displayed eight variations in the eight cases, consisting of two missense mutations (p.Q812E, p.A1363T), five copy number gain mutations and one intron mutation. In the five cases with variations in *ERBB2* , there were five variations, including four missense mutations (p.N125S, p.G603S, p.S310F, p.D1144H) and one frameshift mutation (p.N125S). Furthermore, *BRAF* manifested four variations, including three missense mutations (p.I582V, p.E695Q, p.V600E) and one copy number gain mutation. The concurrent presence of *ALK* -positive NSCLC and *EGFR* mutations is an infrequently observed clinical phenomenon, suggesting the potential for concurrent targeting of *ALK* and *EGFR* as an effective therapeutic approach for these patients. The current study revealed 10 cases where *ALK* fusion co-occurred with *EGFR* mutations, with three cases involving *EML4-ALK* fusion and *EGFR* p.L858R co-mutations, and one case featuring *EML4-ALK* fusion and *EGFR* p.E746_A750del co-mutation ( Fig. 4A ).

Comparatively, when patients with *ALK* fusion-negative NSCLC were examined, *EGFR* alterations were shown to be the most prevalent (51%), followed by *TP53* (42.7%), *KRAS* (11.6%), and *CDKN2A* (11.3%). Other genomic alterations included *PIK3CA* (10.1%), *ERBB2* (7.5%), *MET* (6.7%), *NF1* (5.5%), and *PTEN* (4.3%; Fig. 4B ).

In the present cohort of patients with *ALK* fusion-positive tumor, five patients were identified as carrying germline mutations in five different cancer predisposition genes. These mutations included two pathogenic alterations in *RAD50* (c.1969 + 1G > A) and *BRCA1* (c.3841C > T), two likely pathogenic alterations in *BRCA2* (c.2180C > G) and *FANCL* (c.96 + 2T > A), and one mutation of uncertain significance in *SLX4* (c.2854_2855delGCinsAT). Additional clinical details and the distribution of these germline mutations in the patient cohort are provided in Table 4 .

**Table 4:** Germline mutations in five *ALK* fusion-positive tumors patients

Patient	Sex	Age	Gene	C.	P.	Mut. type	Clinical significance	Evidence source
Pt44	Male	72	RAD50	c.1969 + 1G > A	–	Splice	Pathogenic	ClinVar
Pt45	Female	59	BRCA1	c.3841C > T	p. Q1281 ^*^	Nonsense	Pathogenic likely	ClinVar
Pt46	Female	68	FANCL	c.96 + 2T > A	–	Splice	Pathogenic likely	ClinVar
Pt18	Female	73	BRCA2	c.2180C > G	p.S727 ^*^	Nonsense	Pathogenic	ClinVar
Pt16	Female	69	SLX4	c.2854_2855delGCinsAT	p. A952M	Missense	Uncertain significance	ClinVar

### Age, Sex, and PD-L1 Expression in *ALK* Fusion-Positive Tumors

**Fig. 5:**
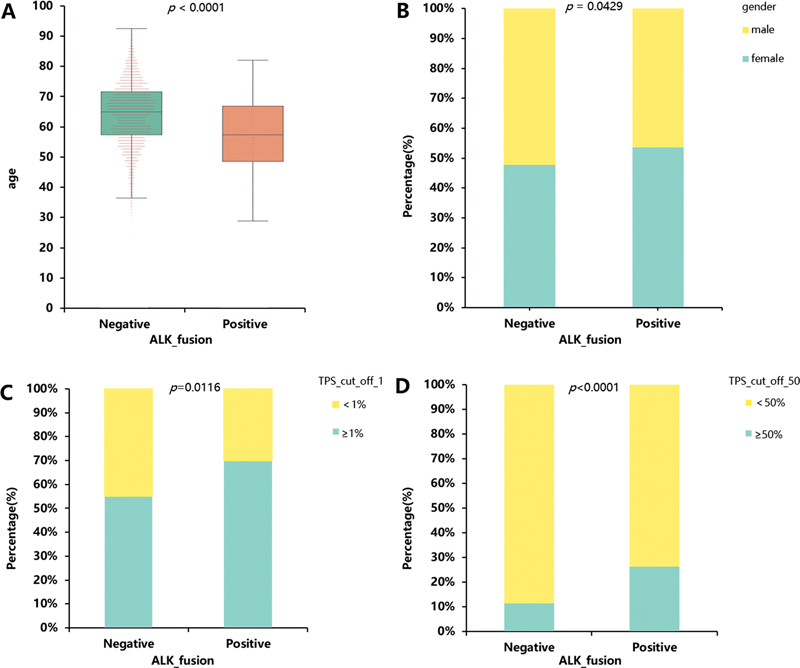
The age, gender and PD-L1 expression of patients between *ALK* fusion-positive group and *ALK* fusion-negative group in nonsmall cell lung cancer (NSCLC). A) The age of patients between ALK+ and ALK− groups. (B) The gender percentage of patients between ALK+ and ALK− groups. (C) The TPS (cutoff = 1%) percentage of patients between ALK+ and ALK− groups. (D) The TPS (cutoff = 50%) percentage of patients between ALK+ and ALK− groups.

Among the 180 patients with *ALK* fusion, there were 93 and 87 female and male patients, respectively, with a median age of 58 years (range, 26–82 years). Notably, patients with NSCLC carrying *ALK* fusion-positive tumors were significantly younger than those with *ALK* fusion-negative tumors ( *p* < 0.0001; Fig. 5A ). This observation aligns with a previous study. 16 Regarding sex differences, a higher relative proportion of women was observed among patients with *ALK* fusion-positive tumors compared with those with *ALK* fusion-negative tumors ( *p* = 0.0429; Fig. 5B ). However, a previous study 17 reported that there were no significant differences in *ALK* fusion between men and women.

The upregulation of the *ALK* fusion protein has been shown to elevate PD-L1 expression, and immunotherapy with anti-PD-1 monoclonal antibodies has demonstrated efficacy in both crizotinib-sensitive and resistant NSCLC cells. 18 Therefore, an assessment of PD-L1 expression within the present cohort was conducted, which consisted of a total of 2,210 eligible patients after the exclusion of those lacking PD-L1 expression data. PD-L1 IHC was performed using the Dako22C3 antibody. In the current comprehensive study, a statistically significant increase was observed in the prevalence of *ALK* -positive test results among patients exhibiting high PD-L1 expression levels, as determined by a 1% cutoff ( *p* = 0.0116; Fig. 5C ). Furthermore, when applying a 50% cutoff, *ALK* fusion-positive tumors exhibited significantly elevated PD-L1 expression compared with *ALK* fusion-negative tumors ( *p* < 0.0001; Fig. 5D ). These findings suggest a potential association between increased PD-L1 expression in *ALK* fusion-positive tumors and poorer progression-free survival (PFS) following TKI therapy.

### Copy Number Variations in Patients with *ALK F* usions

**Fig. 6:**
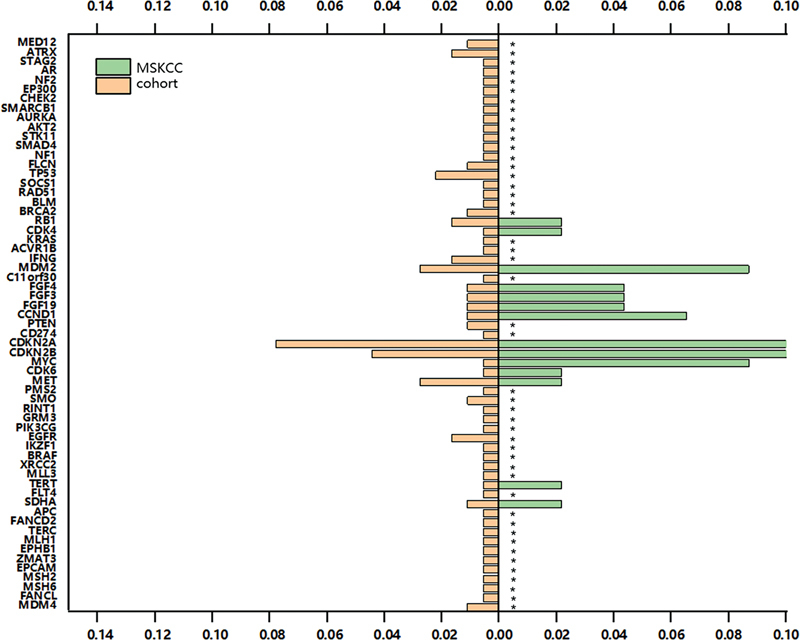
The yellow and green bars represent copy number variation (CNV) events occurring in *ALK* fusion-positive patients in our cohort and Memorial Sloan Kettering Cancer Center (MSKCC) cohort, respectively. *represents the genes not covered in the MSKCC panel.

Copy number variations (CNVs) were identified in 61% (111/180) of the samples in the present study cohort. Notably, approximately 11% of the patients in the cohort exhibited CNVs in *CDKN2A* , a potential candidate contributing to tumorigenesis and disease progression. 18 Furthermore, previous studies have shown association of CNVs in *CDKN2A* , *CDKN2B* , *MCL1* , *MDM2* , and *IRS2* with prognosis. 19 20 21 22 CNVs in *CDKN2A* , *CDKN2B* , *MYC* , *MDM2* , and *CCND1* were also detected in fusion-positive samples from the Memorial Sloan Kettering Cancer Center database. Within the present study cohort, it was observed that CNVs in *CDKN2A* and *CDKN2B* exhibited a notably high frequency ( Fig. 6 ).

## Discussion

In the era of precision medicine, the genomic profiles of patients can play a pivotal role in tailoring treatment strategies. For patients with *ALK* fusion-positive NSCLC, a detailed genomic profile can elucidate the fusion partner and rearranged breakpoint. In the present study, NGS technologies were used to identify *ALK* rearrangement events in 180 Chinese patients with NSCLC. Consistent with previous studies, *EML4* is the most common *ALK* fusion partner, with the fusion occurring in the three predominant variants. 6 Notably, the present study reports the discovery of five novel *ALK* fusion partners: *DAB1* , *KCMF1* , *KIF13A* , *LOC643770* , and *XDH* . This suggests that NGS-based assessment for *ALK* fusions is accurate and comprehensive, offering unique advantages in detecting previously unknown *ALK* fusion partners and precisely identifying breakpoints compared with traditional methods like fluorescence in situ hybridization and IHC.

*EGFR* mutations and *ALK* fusions are the two pivotal driver mutations in NSCLC. Traditionally, *EGFR* mutations and *EML4-ALK* translocations were considered to be mutually exclusive. 23 24 Nonetheless, a growing body of evidence suggests that concurrent mutations, although infrequent, can occur. 25 26 This phenomenon can be attributed to two situations. First, tumor heterogeneity, where distinct tumor cell clones individually carry either an *EGFR* mutation or an *ALK* fusion. 27 Second, the same tumor cell clone harbors both an *EGFR* mutation and an *ALK* rearrangement. 28 29 In the current study, 10 patients with *EGFR/ALK* co-mutations were identified. Among them, three cases involved *EML4-ALK* fusion and *EGFR* p.L858R co-mutations, whereas one case featured *EML4-ALK* fusion and an *EGFR* p.E746_A750del co-mutation. However, there is limited information available regarding the effects of pharmaceutical treatment on these concurrent mutations. A recent study suggested that *EML4-ALK* rearrangements could serve as a rare, acquired resistance mechanism following EGFR-TKI treatment. 30 Nevertheless, there are also studies indicating a more common acquisition of *EGFR* mutations following ALK-TKI therapy. 30

Moreover, Christopoulos et al 31 reported that concurrent *TP53* mutations serve as a robust prognostic indicator in patients with *ALK* fusion-positive NSCLC. 31 The authors further indicated that the *EML4-ALK* fusion variant V3 was linked to a more aggressive phenotype and inferior overall survival (OS) due to the early failure of various therapeutic approaches. Additionally, they observed that patients positive for both V3 and *TP53* alterations faced a notably high risk of death, with an OS of approximately 2 years. In the present dataset of 180 *ALK* fusion-positive samples, *TP53* alterations were the most prevalent co-mutations, occurring in 26.3% of cases. Consequently, these patients may be independently associated with increased metastatic potential, shorter responses to TKI treatment, and poorer OS in *ALK* lung adenocarcinoma. Both of these markers hold the potential to aid in selecting cases for more aggressive management and guiding the development of novel therapeutic strategies.

The data of the present study revealed significantly higher PD-L1 expression in tumors with *ALK* fusions, particularly when using a 5% cutoff compared with fusion-negative tumors. Several studies have demonstrated an association between high tumor PD-L1 expression and poorer PFS in response to ALK-TKIs. 32 33 34 35 36 A prior study indicated that PD-L1 expression status alone did not markedly impact the OS of patients with *ALK* -positive NSCLC. 37 Nevertheless, a previous study found that high baseline PD-L1 expression was associated with shorter OS in *ALK* -rearranged lung adenocarcinoma. 36 In another recent study, patients with high PD-L1 expression were found to exhibit an immunosuppressive status in the tumor microenvironment (TME). The characteristics of the TME may aid in identifying patients who would derive greater benefits from ALK-TKIs. 38

However, the current study also has several limitations. First, complete and detailed patient clinicopathological characteristics, as well as treatment details, including the survival status of all patients with *ALK* fusion-positive NSCLC were not collected. Second, the functional properties of these novel *ALK* fusion proteins and their potential impact on TKI therapy remain unexplored, and further investigation is required. By conducting an analysis of the protein structure and functional sequences of *ALK* , significant *ALK* fusion variants can potentially be identified.

Despite the aforementioned limitations, the present study represents a comprehensive analysis of *ALK* fusions in a substantial cohort of Chinese patients with NSCLC. The current study outcome contributes valuable genomic information for personalized clinical management in the era of precision medicine for patients with *ALK* fusions.
